# Sugar sweetened beverage consumption by Australian children: Implications for public health strategy

**DOI:** 10.1186/1471-2458-11-950

**Published:** 2011-12-22

**Authors:** Katherine Hafekost, Francis Mitrou, David Lawrence, Stephen R Zubrick

**Affiliations:** 1 Telethon Institute for Child Health Research, Centre for Child Health Research, The University of Western Australia, PO Box 855, West Perth WA 6872, Australia

## Abstract

**Background:**

High consumption of sugar sweetened beverages (SSBs) has been linked to unhealthy weight gain and nutrition related chronic disease. Intake of SSB among children remains high in spite of public health efforts to reduce consumption, including restrictions on marketing to children and limitations on the sale of these products in many schools. Much extant literature on Australian SSB consumption is out-dated and lacks information on several key issues. We sought to address this using a contemporary Australian dataset to examine purchase source, consumption pattern, dietary factors, and demographic profile of SSB consumption in children.

**Methods:**

Data were from the 2007 Australian National Children's Nutrition and Physical Activity Survey, a representative random sample of 4,834 Australian children aged 2-16 years. Mean SSB intake by type, location and source was calculated and logistic regression models were fitted to determine factors associated with different levels of consumption.

**Results:**

SSB consumption was high and age-associated differences in patterns of consumption were evident. Over 77% of SSB consumed was purchased via supermarkets and 60% of all SSB was consumed in the home environment. Less than 17% of SSB was sourced from school canteens and fast food establishments. Children whose parents had lower levels of education consumed more SSB on average, while children whose parents had higher education levels were more likely to favour sweetened juices and flavoured milks.

**Conclusions:**

SSB intake by Australian children remains high and warrants continued public health attention. Evidence based and age-targeted interventions, which also recognise supermarkets as the primary source of SSB, are recommended to reduce SSB consumption among children. Additionally, education of parents and children regarding the health consequences of high consumption of both carbonated and non-carbonated SSBs is required.

## Background

There has been a well-documented rise in the prevalence of overweight, obesity and lifestyle related diseases in the Australian population over recent decades [1]. Although recent evidence suggests that the prevalence of overweight and obesity in Australian children has plateaued [2], within the global context the current levels remain disturbingly high. Additionally, the significant associated costs to both the individual and community warrant continued public health focus and development of effective interventions.

Despite much research, and significant public health and media attention, prevention and effective long term treatment of excess weight gain and lifestyle related conditions remains elusive. Although it is likely that these conditions are due to a combination of factors, the rapid increase in their prevalence suggests changing environmental factors may play a significant role. One potential factor, which is temporally associated with the rise in overweight and obesity, is an increased consumption of refined carbohydrate [3].

Sugar sweetened beverages (SSBs) are a significant source of refined carbohydrate in the diet of developed nations such as the United States and Australia [4-7]. Current research has attempted to examine the contribution of SSB consumption towards excess weight gain and disease. Notwithstanding differences in methodology, participants, definitions of SSBs and funding sources [8], well-designed longitudinal and prospective studies typically report positive associations between higher and more regular SSB consumption and detrimental health outcomes [9]. While the exact biological mechanisms which link SSB consumption and weight gain remain unknown, a number of plausible hypotheses have been proposed which explain how energy from SSB may bypass the homeostatic regulatory systems that control appetite and energy intake resulting in increased hunger, reduced satiety and excessive energy consumption. For example, incomplete compensation for calories consumed as liquids, in comparison to isocaloric solids, may contribute to excess energy intake and increased risk of weight gain and the high glycemic load typical of SSBs may reduce satiety and increase risk of disease. Additionally, the unique metabolic pathway of fructose may increase the risk of a number of short and long term negative health outcomes, and some evidence suggests high SSB consumption is linked with other poor dietary patterns which may further increase the risk of weight gain and disease [10-12].

In recognition of the link between SSB and excess weight gain, the Australian Dietary Guidelines [13] recommend limiting the consumption of soft drink and cordial. Health advocacy groups, such as the Australian Medical Association [14] and Public Health Association of Australia [15] have called for a number of additional interventions to reduce SSB intake. Proposed measures include the taxation of nutritionally poor products such as soft drinks with generated revenue to be channelled into preventive programs and healthcare, restriction of the sale of soft drinks in schools, and limitations on the promotion and advertising of these products to children. These strategies mirror international efforts to reduce SSB consumption by children.

Despite the increased public health attention, additional restrictions on marketing, and limitations on the sale of these products in many schools, intake of SSBs by children remains high. Moreover, the extant literature lacks comprehensive descriptions of the nature of SSB consumption in Australia with respect to the source, pattern, demographic and other dietary factors associated with intake. We aim to address this by describing the major sources of SSB, the patterns of consumption, and demographic and dietary factors associated with high and regular consumption in a representative random sample of Australian children. Additionally, we sought to identify potential target areas for future public health intervention.

## Methods

### Data

Data were from the 2007 Australian National Children's Nutrition and Physical Activity Survey (NCNPAS), funded jointly by the Australian Government Department of Health and Aging, Australian Government Department of Agriculture, Fisheries and Forestry and industry group the Australian Food and Grocery Council. The NCNPAS was designed to assess the nutrient intake and physical activity levels of a representative sample of Australian children. Demographic, dietary, physical activity and basic anthropometric data were collected from 4,487 children aged between 2 and 16 years. Interviews were conducted from February through August of 2007.

Food, beverage and supplement intake was collected from participants and caregivers, for children 8 years and younger who required assistance, in two standardised multiple-pass 24 hour recalls. Additional information regarding usual dietary habits, including self- or caregiver reported usual daily intake of fruit and vegetables (number of serves), type of milk consumed, whether salt was added to cooking or meals, and type of salt used (iodised or non-iodised) was collected. This dietary intake information, in addition to socio-demographic data, was collected during a computer assisted personal interview (CAPI) and a second, follow up computer assisted telephone interview (CATI) was completed within 7 to 21 days. Height, weight, and waist circumference data were collected using recognised protocols [16]. The final sample, who provided socio-demographic information and a minimum of one day of dietary recall data, was 4,834. This represented 40% of eligible households. As demographic information was collected at the time of the CAPI, for families who were initially recruited but did not complete the personal interview due to study quotas being filled (n = 1,450) or withdrew part way (n = 502), no demographic information was available. Additionally, as random digit dialling was used in recruitment, there is no information about the characteristics of non-respondents. Further information on study methodology can be found in the NCNPAS User Guide [17].

### Data treatment

#### Beverage classification

For the purpose of this analysis, beverages were classified as follows: water, non-flavoured milk, sugar sweetened beverages (SSB), pure fruit juice (no added sugar), hot tea and coffee (regardless of added sugar), alcohol, artificially sweetened diet beverages, and other (e.g. alcohol and vegetable juice). SSBs were further categorised into carbonated products (including energy drinks), juices with added sugar, cordial (defined as flavoured drink concentrate), sports drinks, milkshakes/smoothies, and flavoured milk. Flavoured milks included both low- and high-fat products as both have substantial sugar content and sugar levels were generally comparable.

#### Location

Location of beverage consumption was provided on the NCNPAS dataset as: home or any other residence, place of purchase, institution (including school and childcare), during transport, leisure activity and other.

#### Source

The source of beverage referred to where the product was purchased and included the following categories: fast food outlets including school canteens, packaged beverages purchased from supermarkets, and fresh products such as homemade juices, milkshakes or smoothies. As the contribution from fresh products was small, for analytic purposes this category was combined with products from unknown sources.

#### Levels of consumption

Children were classified as high, low-moderate or non-consumer of SSB based on their level of intake of SSB during the study period. Children whose SSB intake contributed more than one third of their total daily intake of beverages by volume were classified as high consumers. As the average volume of beverage consumed by children was approximately 1.4 litres per day, this cut-point was equivalent to slightly less than two glasses of SSB and approximately 50 grams of sugar per day. Those who reported no SSB consumption during the study were labelled non-consumers, and the remaining children were considered low-moderate consumers. Additionally, high consumption of each subgroup of SSB was classified separately. Children whose consumption was in the 90th percentile or above for each of the three main categories, carbonated drinks, sweetened juice or flavoured milk, were considered high consumers.

In order to investigate factors associated with regular intake, consumption on both days of dietary data collection was used as a proxy measure of regular SSB consumption. This analysis only included children who provided 2 days of completed dietary recall data (n = 4,633, 95.8%). Children who reported consuming SSB on both recall days were classified as regular consumers (n = 2,331). This classification was used as an additional measure of intake and it was unrelated to overall level of consumption. The majority of regular consumers (74%) were not considered high SSB consumers.

#### Demographic variables

##### Household variables

Household variables included the total annual household income (before tax) at the time of the survey and number of children living in the household. Family type was derived and categorised into original family, step or blended family, one parent family, and other (e.g. multi-generational families). The highest level of education achieved by either parent or caregiver within the household was categorised into university degree, vocational education (TAFE certificate or diploma), year 11-12 schooling, and year 10 schooling or below.

##### Maternal variables

Maternal age at the birth of the study child and breast feeding duration, as recalled by the mother, were included in modelling.

##### Child variables

Study age groups were selected to align with the Nutrient Reference Values for Australia and New Zealand age bands [18]. Birth weight in grams was categorised into low (≤ 2500 g), normal (male > 2500 g-4200 g, female > 2500 g-4000 g) and high (male > 4200 g, female > 4000 g), and current body mass index was derived and compared to age and sex relevant criteria which allowed classification of participants as underweight, normal weight, overweight or obese [19].

#### Dietary variables

Dietary variables were based on the 2-day dietary recall data. Added sugar intake, excluding sugars from beverages and fruit, and saturated fat consumption were calculated as a percentage of total daily energy intakes from dietary recall data.

Average calcium intake was calculated from dietary recall data and compared to age and sex based recommended dietary intakes references. Average daily caffeine intake was similarly calculated and compared to age relevant cut-offs. As no Australian recommendations exist for intake of caffeine by children, values were compared to age-based Canadian recommendations [20]. In addition, parent and child reported usual daily intake of fruit and vegetables, which were collected at the time of the CATI, were included.

### Statistical analysis

All statistical analyses were carried out using SAS Version 9.2 (SAS Institute, Inc., Cary, NC) taking into account population weights and the complex survey design. Population level average intake was calculated using survey weights and associated standard errors were calculated using expansion in Taylor series and the ultimate cluster variance estimate technique [21]. Mean intakes were calculated for beverage category, SSB type, SSB source and location of SSB consumption. T-tests were used to determine the significance of comparisons included in this report.

Six logistic regression models were fitted, adjusting for the complex sample design, to evaluate the associations between demographic and dietary factors and high, no and regular SSB consumption. Factors associated with high consumption of the most commonly consumed sub-groups of SSBs, carbonated beverages, sweetened juices and flavoured milk, were determined. Sports drinks, smoothies/milkshakes and cordials contributed little to overall consumption, and therefore these categories of SSB were not included in sub-group analyses. Variables were eliminated from the final models if statistically non-significant, as determined by Wald's test (alpha = 0.05), and the most parsimonious model reported.

Sensitivity analysis was undertaken to investigate the effect of altering the cut-point used to define high SSB consumption. Logistic regression models were run using alternative cut-points (40, 25 and 20 per cent of daily beverage intake) in order to assess the impact on the strength and direction of results. The average difference between the results of these models was less than 10 per cent, and the use of different cut-points did not change the direction of any results, not did it affect the interpretation of the final models.

## Results

With respect to the general characteristics of the sample, comparisons with the national census figures [22] revealed differences between the study participants and the general Australian population (Table 1). Households participating in the NCNPAS typically had higher levels of education and income.

**Table 1:** Participant Characteristics

		NCNPAS		2006 Census
		
		N	Per cent	Per cent
**Age group**	2-3 years	1191	24.6	12.5
	
	4-8 years	1263	26.1	32.5
	
	9-13 years	1219	25.2	34.1
	
	14-16 years	1161	24.0	20.9

**Sex**	Male	2437	50.4	51.3
	
	Female	2397	49.6	48.7

**SSB consumption**	High	688	14.2	-
	
	Low-moderate	3188	66.0	-
	
	No	958	19.8	-

**BMI category**	Underweight	223	4.5	-
	
	Normal weight	3481	72.0	-
	
	Overweight	800	16.6	-
	
	Obese	330	6.8	-

**Highest Level of Household Education^a^**	University qualification	1029	21.3	26.8
	
	Vocational qualification	1728	35.8	33.0
	
	Year 12 or below	1364	28.2	32.0
	
	Other	713	14.8	8.3

**Household Income ($AUS per week)^b^**	$1-$499	362	7.5	8.8
	
	$500-$1000	967	20.0	21.8
	
	$1000-$2000	1973	40.8	34.9
	
	≥ $2000	1246	25.8	20.7
	
	Other	269	5.6	13.2
	
	Nil/negative	20	0.4	0.7

^a ^Census data includes families with children aged 0-17 years

^b ^Census data includes families with children aged 15 years and below, and families with dependent children aged 15-24 years

Approximately 14% of participants were considered high SSB consumers, 66% were low-moderate consumers and approximately 20% reported no SSB consumption on either study day (Table 1).

The contribution of SSB to total daily energy intake is displayed in Table 2. The proportion of total energy intake from SSB for high consumers was double that of low-moderate consumers (high consumers 14.2%, 95% CI = 13.7-14.7, Low-moderate 6.0%, 95% CI = 5.8-6.2, p < 0.001). Age related differences in the average contribution of SSB to total energy intake were evident. Older children consumed a significantly greater proportion of energy from SSB than younger children (2-3 year olds 4.0%, 95% CI = 3.7-4.3, 14-16 year olds 7.5%, 95% CI = 7.0-7.9, *p *< 0.001).

**Table 2:** Contribution of SSB to Total Daily Energy Intake, by Level of Consumption, Age Group and Sex

Level of SSB Consumption		2-3 years (n = 1191)		4-8 years (n = 1262)		9-13 years (n = 1219)		14-16 years (n = 1161)	
		
	Sex	Per cent	95% CI	Per cent	95% CI	Per cent	95% CI	Per cent	95% CI
**No SSB**	Male	0.0	(0.0-0.0)	0.0	(0.0-0.0)	0.0	(0.0-0.0)	0.0	(0.0-0.0)
	
	Female	0.0	(0.0-0.0)	0.0	(0.0-0.0)	0.0	(0.0-0.0)	0.0	(0.0-0.0)

**Low-moderate**	Male	5.2	(4.7-5.7)	5.9	(5.4-6.3)	6.2	(5.8-6.6)	6.6	(6.1-7.1)
	
	Female	4.6	(4.2-5.0)	5.5	(5.1-6.0)	6.4	(5.9-6.9)	6.8	(6.4-7.3)

**High^a ^**	Male	15.9	(14.0-17.8)	13.5	(12.1-14.9)	13.3	(12.2-14.4)	14.9	(13.7-16.1)
	
	Female	13.3	(11.6-15.0)	13.1	(11.9-14.4)	14.1	(12.8-15.3)	16.4	(14.7-18.1)

**Total**	Male	4.2	(3.7-4.6)	5.6	(5.1-6.1)	6.7	(6.2-7.2)	7.6	(7.0-8.2)
	
	Female	3.8	(3.4-4.2)	5.1	(4.7-5.6)	6.7	(6.1-7.2)	7.4	(6.6-8.1)

^a ^High consumption: > 1/3 of average daily beverage intake from SSB

Age-associated differences in the contribution of SSB sub-categories to total intake were evident (Table 3). The main source of SSB in the diet of younger children was sweetened juice (2-3 year olds 39.9%, 95% CI = 36.7-43.2). For each successive age group we observed a greater mean contribution from carbonated beverages and, in children over 9 years of age, carbonated drinks were the primary type of SSB consumed (14-16 year olds 41.7%, 95% CI = 38.6-44.8, *p *< 0.001). There was no reduction in the volume of non-carbonated SSBs consumed among the older age groups to compensate for the higher carbonated SSB consumption (Table 4).

**Table 3:** Mean Contribution of SSB Subcategories to Total SSB Intake, by Age Group

Beverage Classification (SSBs)	2-3 years (n = 836)		4-8 years (n = 1012)		9-13 years (n = 1055)		14-16 years (n = 972)	
	
	Per cent	95% CI	Per cent	95% CI	Per cent	95% CI	Per cent	95% CI
**Carbonated soft drink**	15.0	(12.8-17.3)	23.4	(20.9-25.8)	38.4	(35.7-41.1)	41.7	(38.6-44.8)

**Juice - added sugar**	39.9	(36.7-43.2)	34.7	(32.0-37.4)	23.9	(21.5-26.2)	20.9	(18.5-23.3)

**Cordial**	19.9	(16.3-21.6)	12.8	(10.8-14.9)	11.6	(9.6-13.5)	10.0	(8.0-11.9)

**Sports drink**	0.3	(0.0-0.6)	0.9	(0.4-1.4)	3.1	(2.1-4.1)	3.5	(2.3-4.7)

**Flavoured milk**	22.1	(19.4-24.7)	24.1	(21.5-26.6)	19.6	(17.0-22.1)	21.2	(18.6-23.8)

**Milkshake/Smoothie**	3.8	(2.5-5.1)	4.1	(2.9-5.3)	3.5	(2.6-4.5)	2.8	(2.0-3.5)

**Table 4:** Mean Volume (mls) of SSB Subcategories Consumed, by Age Group

Beverage Classification (SSBs)	2-3 years (n = 836)		4-8 years (n = 1012)		9-13 years (n = 1055)		14-16 years (n = 972)	
	
	Volume(mls)	95% CI	Volume (mls)	95% CI	Volume (mls)	95% CI	Volume (mls)	95% CI
**Carbonated soft drink**	56.5	(46.7-66.3)	134	(116 - 152)	305	(276-333)	426	(386-467)

**Juice - added sugar**	145	(129-161)	176	(159-192)	157	(140-175)	175	(149-201)

**Cordial**	27.7	(22.9-32.5)	28.7	(24.3-33.1)	40.3	(32.8-47.9)	38.4	(31.0-45.8)

**Sports drink**	1.2	(0.0-2.6)	8.4	(3.6-13.2)	30.1	(19.7-40.6)	38.2	(26.8-49.5)

**Flavoured milk**	38.8	(31.1-46.6)	74.3	(63.5-85.2)	84.3	(71.6-96.9)	131	(114-149)

**Milkshake/Smoothie**	12.9	(8.4-17.5)	21.8	(15.6-28.0)	28.9	(21.5-36.4)	25.2	(18.2-32.2)

The majority of SSB was consumed at home (2-3 year olds 81.1%, 95% CI = 78.8-83.5, 4-8 year olds 67.2%, 95% CI = 64.4-70.0, 9-13 year olds 66.1%, 95% CI = 63.3-68.8, 14-16 year olds 63.1%, 95% CI = 60.6-65.6). The proportion of SSB consumed at the place of purchase, in institutions and during leisure activities and transport was higher in older age groups. However, even amongst the oldest children who reported the greatest variation in the location of consumption, over 60 per cent of SSB was still consumed in the home (Table 5).

**Table 5:** Proportion of SSB Consumed at Home, Place of Purchase, Institution and Other Locations

Location	2-3 years (n = 836)		4-8 years (n = 1012)		9-13 years (n = 1055)		14-16 years (n = 972)	
	
	Per cent	95% CI	Per cent	95% CI	Per cent	95% CI	Per cent	95% CI
**Home**	81.1	(78.8-83.5)	67.2	(64.4-70.0)	66.1	(63.3-68.8)	63.1	(60.6-65.6)

**Place of Purchase**	9.4	(7.5-11.3)	11.5	(9.5-13.5)	11.4	(9.5-13.3)	13.0	(11.0-14.9)

**Institution**	3.5	(2.4-4.6)	13.6	(11.6-15.6)	10.6	(8.9-12. 2)	11.4	(9.6-13.2)

**Other**	5.3	(4.0-6.7)	7.4	(6.1-8.7)	11.8	(10.1-13.4)	12.4	(10.6-14.1)

Supermarket purchased products were the main source of SSB in all age groups (2-3 year olds 87.9%, 95% CI = 85.9-90.0, 4-8 year olds 77.2%, 95% CI = 74.9-79.5, 9-13 year olds 76.2%, 95% CI = 73.8-78.6, 14-16 year olds 74.38%, 95% CI = 72.0-76.8). Although the average contribution of SSB from fast food sources was higher in successive age groups, it remained relatively small (2-3 year olds 9.3%, 95% CI = 7.3-11.2, 14-16 year olds 21.1%, 95% CI = 18.7-23.4, *p *< 0.001) (Table 6). Of note, as a result of difficulty in determining whether beverages consumed in institutions were supermarket purchased and brought from home or purchased at the institution, many SSBs consumed in this location were coded as from an unknown source (52%).

**Table 6:** Proportion of SSB from Supermarket, Fast Food and Unknown Place of Purchase

Source	2-3 years (n = 836)		4-8 years (n = 1012)		9-13 years (n = 1055)		14-16 years (n = 972)		All (n = 3875)	
	
	Per cent	95% CI	Per cent	95% CI	Per cent	95% CI	Per cent	95% CI	Per cent	95% CI
**Supermarket**	87.9	(85.9-90.0)	77.2	(74.9-79.5)	76.2	(73.8-78.6)	74.4	(72.0-76.8)	77.4	(76.1-78.8)

**Fast Food**	9.3	(7.3-11.2)	14.5	(12.4-16.5)	18.3	(16.1-20.5)	21.1	(18.7-23.4)	16.7	(15.4-18.0)

**Unknown**	3.2	(1.9-4.6)	8.9	(7.0-10.8)	5.8	(4.5-7.0)	4.9	(3.5-6.3)	6.3	(5.4-7.2)

High consumption of SSB, defined as consumption which was greater than a third of daily beverage intake, was associated with older age groups, male gender, lower levels of household education and a number of markers of poor dietary patterns including lower intake of fresh fruit and vegetables, and high caffeine consumption (Table 7). Conversely, no SSB consumption was associated with younger age groups, higher levels of household education and markers of healthy dietary patterns. Both high and non-consumers were more likely to be below the recommended dietary intake of calcium than low-moderate SSB consumers.

**Table 7:** Factors Associated with High, No and Regular SSB Consumption

		High SSB Consumption^a^		No SSB Consumption^b^		Regular SSB Consumption^c^	
		
		OR	95% CI	OR	95% CI	OR	95% CI
**Age group**	2-3 years	0.31	(0.23-0.42)	3.32	(2.58-4.28)	0.41	(0.33-0.51)
	
	4-8 years	0.65	(0.51-0.83)	1.82	(1.46-2.25)	0.69	(0.58-0.84)
	
	9-13 years^d^	1.00					
	
	14-16 years	1.18	(0.94-1.49)	1.18	(0.93-1.51)	0.90	(0.74-1.08)

**Sex**	Female^d^	1.00		^e^			
	
	Male	1.22	(1.01-1.48)			1.21	(1.06-1.39)

**Highest Level of Household Education**	University qualification^d^	1.00					
	
	Vocational qualification	1.68	(1.24-2.27)	0.57	(0.47-0.69)	1.38	(1.17-1.64)
	
	Year 11-12	1.79	(1.30-2.46)	0.68	(0.54-0.86)	1.36	(1.13-1.63)
	
	≤ Year 10	2.11	(1.32-3.36)	0.47	(0.28-0.78)	1.84	(1.24-2.74)
	
	Unknown	1.96	(1.38-2.78)	0.52	(0.39-0.70)	1.81	(1.47-2.25)

**Number of Children in Household**	≥ 4^d^	1.00		^e^			
	
	3	1.19	(0.78-1.80)				
	
	2	1.01	(0.73-1.63)				
	
	1	1.63	(1.12-2.39)				

**Daily Vegetable Intake**	≥ 4 serves	0.78	(0.61-1.01)	1.49	(1.19-1.88)	^e^	
	
	2-3 serves^d^	1.00					
	
	< 2 serves	1.29	(1.03-1.61)	0.93	(0.76-1.15)		

**Daily Fruit Intake**	> 2 serves	0.75	(0.58-0.97)	1.13	(0.92-1.38)	1.41	(1.18-1.69)
	
	2 serves^d^	1.00					
	
	< 2 serves	1.19	(0.92-1.54)	0.78	(0.63-0.98)	0.85	(0.72-1.00)

**Calcium Intake^f^**	≥ RDI^d^	1.00					
	
	< RDI	1.32	(1.04-1.67)	1.35	(1.11-1.34)	0.70	(0.58-0.84)

**Caffeine Intake^g^**	≤ RDI*	1.00		^e^			
	
	> RDI	2.32	(1.59-3.38)				

^a ^High (> 1/3 of average daily beverage intake) vs. low-medium and no consumers

^b ^No vs. low-medium and high consumers

^c ^Regular (Consumed SSB on both study days) vs. irregular (SSB consumption on 1 or no days)

^d ^Reference category

^e ^Non-significant variables tested but eliminated from the final models (p > 0.05). Additional variable tested but eliminated from all models included location (rural or metropolitan), mother born overseas, birth weight, breastfeeding duration and BMI category.

^f ^RDI cut-offs for calcium: 1-3 years < 500 mg/day, 4-8 years < 700 mg/day, 9-11 years < 1,000 mg/day, 12-13 years < 1,300 mg/day, 14-18 years < 1,300 mg/day

^g ^High caffeine: 2-6 years > 45 mg per day, 7-9 years > 62.5 mg per day, 10-12 years > 85 mg per day, 13-16 years > 95 mg per day

Regular intake of SSB, defined by consumption of SSB on both study days, was associated with older age groups, male gender, lower levels of household education, and low daily intake of fruit (Table 7). There was no significant relationship between regular consumption and vegetable or caffeine intake. Additionally, regular SSB consumers were less likely to be below the recommended dietary intake for calcium than irregular consumers.

Factors associated with high consumption of the three main sub-groups of SSB differed (Table 8). Whilst high intake of carbonated SSB was significantly associated with lower levels of household education, intakes of sweetened juice and flavoured milk did not vary with levels of household education. Similarly suggestive of an association between parental education and dietary choices, we observed that high consumption of carbonated drinks was associated with lower intake of fruit, but high consumption of flavoured milk and sweetened juice were not.

**Table 8:** Factors Associated with High Consumption of Carbonated Drinks, Sugar Sweetened Juice and Flavoured Milk

		Carbonated Drinks^a^		Sweetened Juice^a^		Flavoured Milk^a^	
		
		OR	95% CI	OR	95% CI	OR	95% CI
**Age group**	2-3 years	0.04	(0.02-0.09)	0.48	(0.35-0.67)	0.12	(0.08-0.20)
	
	4-8 years	0.20	(0.14-0.29)	0.79	(0.58-1.08)	0.45	(0.33-0.61)
	
	9-13 years^b^	1.00					
	
	14-16 years	1.25	(1.06-1.73)	1.22	(0.93-1.61)	1.37	(1.08-1.74)

**Sex**	Female^b^	1.00				^c^	
	
	Male	1.92	(1.49-2.47)	1.46	(1.17-1.83)		

**Highest Level of Household Education**	University qualification^b^	1.00		^c^		^c^	
	
	Vocational education	1.80	(1.25-2.59)				
	
	Year 11 or 12	1.71	(1.18-2.48)				
	
	Year 10 or below	2.21	(1.27-3.83)				
	
	Don't know	2.24	(1.53-3.28)				

**Daily Fruit Intake**	> 2 serves	0.70	(0.52-0.93)	^c^		^c^	
	
	2 serves^b^	1.00					
	
	< 2 serves	1.28	(0.98-1.66)				

**Calcium Intake^d^**	≥ RDI^b^			^c^		1.00	
	
	< RDI					0.37	(0.29-0.46)

**Caffeine Intake^e^**	No^b^	1.00					
	
	Yes	3.15	(2.12-4.66)	^c^		2.32	(1.58-3.41)

^a ^High consumption: ≥ 90^th ^percentile of intake

^b ^Reference category

^c ^Non-significant variables tested but eliminated from the final models (p > 0.05). Additional variable tested but eliminated from all models included location (rural or metropolitan), mother born overseas, birth weight, breastfeeding duration and BMI category.

^d ^RDI cut-offs for calcium: 1-3 years < 500 mg/day, 4-8 years < 700 mg/day, 9-11 years < 1,000 mg/day, 12-13 years < 1,300 mg/day, 14-18 years < 1,300 mg/day

^e ^High caffeine: 2-6 years > 45 mg per day, 7-9 years > 62.5 mg per day, 10-12 years > 85 mg per day, 13-16 years > 95 mg per day

## Discussion

SSBs contributed a substantial amount of energy to the diet of Australian children with mean intakes ranging from 4 per cent in children 2-3 years old to 7.5 per cent in 14 to 16 year olds. These values are lower than those reported for US children (2-18 years) whose SSB intake is approximately 10 per cent of their energy intake [23]. However, given the growing evidence which suggests biological mechanisms linking high and regular consumption of SSBs with negative short and long term health outcomes [8], effective public health intervention to reduce levels of intake by Australian children is recommended.

The majority of SSB in the diet of children in all age groups was sourced from supermarkets and consumed at home. Only a small percentage of SSBs were purchased from fast food sources. Even among the 14-16 year age group, who typically would have a higher level of independence from parents and some discretionary spending compared with younger children, around 75 per cent of their SSB intake was purchased from supermarkets. These findings are similar to those of Wang and colleagues who reported, in a study of US children, 55 to 70 per cent of the calories from SSBs were consumed in the home environment [24]. Although public health strategies and interventions have traditionally focused on fast food sources of SSBs the results of this study suggest that in order to significantly reduce levels of intake by children aged 2-16 years, future strategies should shift their focus to supermarket purchased SSBs and at home consumption.

The variation in the pattern of SSB consumption by age is noteworthy. Of particular interest was the high consumption of sweetened juice by the 2-3 year old children. Similarly, Wang and colleagues reported, in US children, the main type of SSB consumed by children aged 2-5 years was sweetened fruit punch and fruit juice [24]. During a critical period of growth and development the high intake of sugary products is concerning. In addition to the early increased risk of weight gain and associated chronic disease, intake of SSBs at a young age has been linked to lower intake of milk and, as a result, lower intake of calcium, riboflavin, vitamin A and phosphorus [25]. Further, high intake of SSBs has been associated with dental caries [26], poor growth [27] and digestive issues in very young children [28]. In addition to the direct health consequences, there is some evidence to suggest that early food experiences influence later on-going food preferences and dietary patterns [29,30]. At a young age when parents are likely to have almost complete control over a child's diet, replacing SSBs with unsweetened milk or water may be relatively easy. Further, the development of public health strategies to educate parents about the health implications of high consumption of sweetened fruit juice should be a priority. The differences in patterns of SSB consumption between age groups suggests that focused, age relevant interventions to target the main types, sources and locations of SSB intake is likely to improve the effectiveness of strategies.

The demographic and dietary factors associated with high consumption of carbonated SSBs and non-carbonated SSBs differed. While high consumption of carbonated drinks was related to a number of markers of unhealthy dietary patterns and lower levels of household education, non-carbonated SSBs were not. These findings are similar to those of Ranjit and colleagues who reported that high carbonated beverage consumption was associated with poor dietary and physical activity patterns in US children, while consumption of non-carbonated drinks was associated with positive health behaviours [31]. The variation in factors associated with carbonated and non-carbonated SSBs suggests differences in public perception. Non-carbonated SSBs are typically marketed based on health-related claims, or as functional beverages which contribute to a healthy balanced diet and active lifestyle. In contrast, carbonated SSBs have been heavily targeted by public health advocates. These easily identifiable products are well recognised as 'junk foods' with little nutritional value and consumption of these products is likely to be limited in health conscious families. Additionally, the Australian Dietary Recommendations [13] and Australian Guide to Healthy Eating [32], which mirror the Dietary Guidelines for Americans, suggest limiting intakes of soft drinks, cordials and/or sweetened drinks [13] but do not make explicit whether flavoured milks and sugar sweetened juices are included within this recommendation. Therefore a key step in reducing the intake of SSB appears to be education of the population regarding the health consequences of high and regular consumption of both carbonated SSBs and non-carbonated SSBs, and the role of these products in a healthy and balanced diet. Future public health guidelines should make explicit appropriate intakes and serving sizes for children and make clear recommendations for both carbonated and non-carbonated products.

Using the various definitions of high and regular intake we consistently found SSB intake to be associated with markers of poor dietary patterns. For example, high intake of caffeine, and lower intake of calcium, fruit and vegetables. This finding is supported by previous literature linking SSB consumption with unhealthy dietary habits [10]. However, in contrast with much of the previous literature [8,9] there was no significant association between high or regular consumption of SSB and likelihood of being overweight or obese. This may be due to the cross-sectional study design. As reported dietary patterns are likely to be influenced by current weight status, drawing statistically valid and meaningful inferences from cross sectional data regarding the relationship between SSB consumption and risk of weight gain is impossible.

Growing evidence supports a mechanistic link between the high sugar, and specifically high fructose, content of SSBs and the risk of chronic disease and excess weight gain. The unique metabolic and hormonal effects of fructose are linked to a number of adverse short-term effects including enhanced de novo lipogenesis and triglyceride production resulting in dyslipidaemia, increased systolic blood pressure, reduced insulin and leptin sensitivity, impaired appetite control and visceral adiposity [33-36]. These conditions may contribute to an increased risk of longer term chronic health conditions such as type 2 diabetes, cardiovascular disease, cardio-renal disease, and obesity [37,38]. As common caloric sweeteners such as sucrose, high fructose corn syrup and fruit juice concentrate have relatively high concentrations of fructose they have similar metabolic effects [12]. Therefore, increasing public awareness of the dangers associated with high intake of caloric sweeteners in the diets of children should be of great concern to public health advocates.

The current analysis is limited by its reliance on 2-day dietary recall, a cross sectional study design and the potential for misclassification of beverage type or source. As individual consumption was based on 2 days of dietary recall it is possible that individual consumption on the survey days was not representative of their typical consumption patterns, in particular, for children who did not consume SSBs. However, as the survey days were randomly selected for each survey child, population estimates of average consumption of SSB should be unbiased. As data were self-reported, actual consumption may vary from recorded intakes. Further, the involvement of the parent or caregiver in dietary data collection in younger children (2-8 years) may have potentially influenced the accuracy of results. However, these biases are likely to underestimate the consumption of unhealthy foods such as SSBs and therefore reported intakes are likely to be biased towards the null, leading to an underestimation of the reported associations. The cross-sectional design means it is impossible to associate current consumption with the development of long term health outcomes such as obesity or chronic disease. Due to the use of random digit dealing for participant recruitment no demographic information was available for non-respondents. As a result, differences between study participants and non-respondents could not be determined and potential bias between these groups could not be assessed. However, households participating in NCNPAS typically had slightly higher levels of education and income in comparison to the general Australian population, and this could potentially bias results and limit the generalizability of the research.

## Conclusions

These findings reveal opportunities to close possible gaps in public understanding of the role of both carbonated and non-carbonated SSBs in a healthy diet. Additionally, they highlight the need for evidence based, and age relevant public health interventions which target the primary sources and location of SSB consumption in order to effectively reduce levels of intake by Australian children. Finally, the importance of periodic monitoring of child health and nutrition status, to allow public health strategies to remain effective and relevant to the changing needs of the population, is clear.

## Competing interests

The authors declare that they have no competing interests.

## Authors' contributions

KH, FM and DL conceived the original idea for the study. All authors contributed to the development of the study methodology. KH undertook the data analysis and wrote the first draft of manuscript. All authors contributed to writing the paper, and approved the final manuscript.

## Pre-publication history

The pre-publication history for this paper can be accessed here:

http://www.biomedcentral.com/1471-2458/11/950/prepub
